# Identification and characterization of a novel human hepatocellular carcinoma-associated gene

**DOI:** 10.1054/bjoc.2001.2059

**Published:** 2001-10

**Authors:** Z-X Wang, H-Y Wang, M-C Wu

**Affiliations:** The Department of General Surgery of the Jingcheng Hospital, No 98, Xiaoxihu West Street, Block Qilihe, Lanzhou, Gansu Province, 730050, PR China; Eastern Hepatobilliary Surgical Hospital, The Second Military Medical University, Shanghai, 200438, PR China

**Keywords:** hepatocellular carcinoma, differential display, gene clone, expression

## Abstract

To investigate liver cancer-associated genes and to explore the molecular basis of liver cancer genesis, we have cloned a novel hepatocellular carcinoma (HCC)-related gene with a transcript of 2520 base pairs in length named HCCA2 by mRNA differential display polymerase chain reaction (DDPCR) and screening a placenta cDNA library. No significant homologous protein with known genes was found. Western blot analysis showed that HCCA2 could be expressed in transfected 293 cells. Northern hybridization analysis showed that HCCA2 mRNA was expressed in 79% (34/43) patients with HCC, most of whom had significantly high expression in HCC tissues, while not expressed in corresponding noncancerous liver tissues. The clinical pathological data showed that the HCCA2 was significantly associated with the invasion of tumour capsule (*P*= 0.0007) and the expression of ki-67 protein (*P*= 0.0022). Immunohistochemical staining confirmed that the HCCA2 protein was localized in cytoplasm of liver cancer tissues. According to amino acid analysis of the protein and its localization, it may play a role in a cascade of intracellular signal transduction because the protein was characterized with two Src homology 3 (SH3) binding-domains and several functional motifs of phophorylation. © 2001 Cancer Research Campaign  http://www.bjcancer.com


*British Journal of Cancer* (2001) **85**(8), 1162–1167

© 2001 Cancer Research Campaign doi: 10.1054/ bjoc.2001.2059, available online at http://www.idealibrary.com on IDEAL
http://www.bjcancer.com

### Identification and characterization of a novel human hepatocellular carcinoma-associated gene

**Z-X Wang[1], H-Y Wang[2] and M-C Wu[2]**

[1]The Department of General Surgery of the Jingcheng Hospital, No 98, Xiaoxihu West Street, Block Qilihe, Lanzhou, Gansu Province, 730050, PR China;
[2]Eastern Hepatobilliary Surgical Hospital, The Second Military Medical University, Shanghai 200438, PR China

**Summary** To investigate liver cancer-associated genes and to explore the molecular basis of liver cancer genesis, we have cloned a novel hepatocellular carcinoma (HCC)-related gene with a transcript of 2520 base pairs in length named HCCA2 by mRNA differential display polymerase chain reaction (DDPCR) and screening a placenta cDNA library. No significant homologous protein with known genes was found. Western blot analysis showed that HCCA2 could be expressed in transfected 293 cells. Northern hybridization analysis showed that HCCA2 mRNA was expressed in 79% (34/43) patients with HCC, most of whom had significantly high expression in HCC tissues, while not expressed in corresponding noncancerous liver tissues. The clinical pathological data showed that the HCCA2 was significantly associated with the invasion of tumour capsule ($P$ = 0.0007) and the expression of ki-67 protein ($P$ = 0.0022). Immunohistochemical staining confirmed that the HCCA2 protein was localized in cytoplasm of liver cancer tissues. According to amino acid analysis of the protein and its localization, it may play a role in a cascade of intracellular signal transduction because the protein was characterized with two Src homology 3 (SH3) binding-domains and several functional motifs of phophorylation. © 2001 Cancer Research Campaign http://www.bjcancer.com

**Keywords**: hepatocellular carcinoma; differential display; gene clone; expression

Primary hepatocellular carcinoma is the most common form of primary liver malignancy (Bain and McMaster, 1997; Johnson, 1997), and is among the 10 most common tumours (Sherman, 1995; Duvoux, 1998) in the world. In China, it is a main fatal malignancy. The annual morbidity is at least about 100 000 and the mortality 110 000 in China (Fujimoto et al, 1994). Chronic hepatitis B or C viral infection appears to be the most important risk factor for HCC (Wallner et al, 1994). Also, chemical carcinogens (e.g. aflatoxins and mycotoxins), haemochromatosis and other chronic liver diseases have proved to be closely associated with HCC in China (Fujimoto et al, 1994; Xu et al, 1996). During the long period of chronic liver lesion, inflammation and liver cell turnover, hepatocyte genomic errors accumulate and HCC eventually develop as an end-stage of the chronic liver disease. The molecular events for HCC development are very complex, and HCC has proved to be genetically heterogenous neoplasms (Hui and Makuuchi, 1999; Kondoh et al, 1999; Schlott et al, 1999). There have been at least 30 species of related genes cloned and characterized, and such findings are still increasing (Irene, 1998; Ozturk, 1999). The known genes include several growth factors and their receptors, cell cycle regulators, oncogenes and tumour suppressor genes (Hui and Makuuchi, 1999). But to date, the identified genes have not yet fully disclosed the mechanisms of HCC (Johnson, 1997; Hui and Makuuchi, 1999). Theoretically, there should still be many other genes involved in the development of HCC.

In the attempt to identify HCC-susceptible genes, differential display method was employed in this study (Liang and Pardee, 1992; Liang et al, 1993; Zhang et al, 1996). For an analysis of altered expression genes between HCC tissues and their surrounding liver tissues, we isolated a novel gene termed HCCA2 with a full length of cDNA. We compared HCCA2 expression in HCC with the pathological parameters to probe its potential biological role. Also, we raised antibodies for HCCA2 and investigated its immunohistochemical characterization.

*Received 6 February 2001*
*Revised 30 May 2001*
*Accepted 2 July 2001*

*Correspondence to:* Z-X Wang

### MATERIALS AND METHODS

### Patients and specimens

Primary hepatocellular carcinoma and their corresponding noncancerous liver tissues were obtained from 43 patients who had received surgical resection at the Eastern Hepatobilliary Surgical Hospital of The Second Military Medical University, Shanghai, China. No patients had received preoperative or adjuvant chemotherapy or radiotherapy. Maximum tumour diameter was measured macroscopically in fresh specimens. For RNA analysis, HCC tissues and surrounding liver tissues were separately cut and stored in liquid nitrogen immediately after surgery. These included 42 male and 1 female patients with a median age of 50 years (range 24–72 years). 36 (83.7%) patients had serological evidence of hepatitis B virus infection. The serum AFP level was above 25 µg $L^{-1}$ in 24 cases (55.8%). The tumour size was smaller than 5 cm (small HCC) in 13 patients and larger than 5 cm in 30. Histologically, 41 patients (95.3%) were accompanied by cirrhosis. The 43 tumours comprised 5 well differentiated (Edmondson's grades I & II) and 38 poorly differentiated (Edmondson's grades III & IV) HCCs. Macroscopic portal vein tumour spread was found in 3 patients, and microscopic surrounding liver vascular cancer thrombi were found in 26. Gross and microscopic intrahepatic adjacent small satellite nodule

**Table 1** Details of HCCA2 expression in 43 cases of hepatocellular carcinoma

| Case no. | Sex | Age (yr) | Tumour size (cm) | Tumour differentiation | Serum AFP | HBsAg | Histology of non-tumourous liver | Capsule invasion | Cancer thrombi | Satellite lesion | ki-67 protein | HCCA2 |
|---|---|---|---|---|---|---|---|---|---|---|---|---|
| 1 | M | 65 | 6 | Poor | 3.3 | – | Cirrhosis | + | + | – | – | + |
| 2 | M | 42 | 8.2 | Poor | >1000 | + | Cirrhosis | – | + | + | – | – |
| 3 | M | 38 | 9 | Poor | >1000 | + | Cirrhosis | – | – | + | + | + |
| 4 | M | 64 | 16 | Poor | 5.9 | – | Cirrhosis | – | + | + | + | + |
| 5 | M | 66 | 4 | Well | 8.9 | + | Cirrhosis | – | – | – | – | – |
| 6 | M | 28 | 4 | Poor | 7.7 | + | Cirrhosis | + | + | + | – | + |
| 7 | M | 61 | 5 | Poor | 0.4 | – | Normal | + | – | + | + | + |
| 8 | M | 60 | 10 | Poor | >1000 | – | Cirrhosis | + | + | + | + | + |
| 9 | M | 62 | 11 | Well | >1000 | – | Cirrhosis | + | – | + | – | + |
| 10 | M | 51 | 8 | Poor | >1000 | + | Cirrhosis | + | + | + | + | + |
| 11 | M | 41 | 8 | Poor | >1000 | + | Cirrhosis | + | – | – | + | + |
| 12 | M | 46 | 4.2 | Poor | 9.6 | + | Cirrhosis | + | + | – | + | + |
| 13 | M | 65 | 7.4 | Well | 6 | – | Normal | – | + | – | + | – |
| 14 | M | 24 | 12 | Poor | 37.7 | + | Cirrhosis | + | + | + | + | + |
| 15 | M | 43 | 5 | Poor | 414 | + | Cirrhosis | + | +* | + | + | + |
| 16 | M | 50 | 25 | Poor | 122.9 | + | Cirrhosis | + | + | + | + | + |
| 17 | M | 57 | 6 | Poor | 215 | + | Cirrhosis | + | + | + | + | +[a] |
| 18 | M | 37 | 12 | Poor | 810 | + | Cirrhosis | + | + | – | + | + |
| 19 | M | 58 | 10 | Poor | >1000 | + | Cirrhosis | + | + | + | + | + |
| 20 | M | 47 | 8 | Poor | 9.8 | + | Cirrhosis | + | +* | + | + | + |
| 21 | M | 54 | 3.9 | Poor | 6.0 | + | Cirrhosis | – | – | + | – | – |
| 22 | M | 49 | 10.3 | Poor | >1000 | + | Cirrhosis | + | + | + | + | – |
| 23 | M | 49 | 6.9 | Poor | 33.9 | + | Cirrhosis | – | + | + | – | – |
| 24 | M | 51 | 4 | Well | 28.9 | + | Cirrhosis | – | – | – | – | – |
| 25 | M | 49 | 5 | Poor | 211 | + | Cirrhosis | + | – | + | – | + |
| 26 | M | 54 | 15 | Poor | >1000 | + | Cirrhosis | – | – | – | + | + |
| 27 | M | 33 | 9 | Poor | 7.6 | + | Cirrhosis | + | + | + | + | + |
| 28 | M | 40 | 5 | Poor | 3.1 | + | Cirrhosis | + | + | + | + | + |
| 29 | M | 69 | 6 | Poor | 44.2 | + | Cirrhosis | – | + | + | – | – |
| 30 | M | 44 | 12 | Poor | >1000 | + | Cirrhosis | + | – | + | + | + |
| 31 | M | 45 | 10 | Poor | >1000 | + | Cirrhosis | + | – | + | + | + |
| 32 | M | 44 | 4.2 | Poor | 8.8 | + | Cirrhosis | + | – | – | + | + |
| 33 | M | 39 | 3.5 | Poor | 3.9 | + | Cirrhosis | + | – | + | + | + |
| 34 | M | 41 | 4.5 | Poor | 16.3 | – | Cirrhosis | + | + | + | + | – |
| 35 | M | 44 | 9 | Poor | 6.3 | + | Cirrhosis | + | – | – | + | + |
| 36 | F | 65 | 6 | Poor | >1000 | + | Cirrhosis | + | + | + | + | + |
| 37 | M | 55 | 7 | Poor | >1000 | + | Cirrhosis | + | +* | + | + | +[a] |
| 38 | M | 52 | 9 | Poor | >1000 | + | Cirrhosis | – | – | – | + | + |
| 39 | M | 51 | 9.2 | Poor | >1000 | + | Cirrhosis | + | + | + | + | + |
| 40 | M | 48 | 4 | Poor | 23 | + | Cirrhosis | – | + | + | + | + |
| 41 | M | 64 | 9 | Poor | 8.5 | + | Cirrhosis | + | + | + | + | + |
| 42 | M | 58 | 9 | Well | 14.3 | + | Cirrhosis | + | – | – | + | + |
| 43 | M | 72 | 6.5 | Poor | 25 | + | Cirrhosis | + | – | – | + | + |

M = Male, F = Female; AFP, α-fetoprotein in $\mu g\ l^{-1}$; HBsAg, hepatitis B surface antigen; *had macroscopic portal vein tumour thrombi; [a]had low levels expression of HCCA2 in surrounding liver tissues.

lesions were found in 30, and tumour capsule invasion of liver cancer was founded in 31 (Table 1). Fetal tissues of liver and brain and lung and spleen were obtained from fetuses and newborns during autopsy. Adult normal tissues were obtained from a healthy young man having died of a traffic accident.

### RNA extraction and differential display

Total RNA of human HCC tissues and the paired non-tumourous liver from each patient, and from a series of normal adult tissues and fetal tissues was extracted by the guanidinium isothiocyanate extraction method (Chomczinski and Sacchi, 1987). The differential display method was performed as described previously (Liang and Pardee, 1992; Liang et al, 1993; Zhang et al, 1996). DDPCR fragments were then reamplified by the same primers of the anchoring oligo (dT) primer T12CA (5′-TTTTTTTTTTTTCA-3′) and an arbitrary 10-base olignucleotide A2 (5′-AATCGGGCTG-3′), separated on a 1.6% argarose gel, purified by QIAEX II gel extraction kit (QIAGEN, Hilden, Germany), and subcloned into the pGEM-T vector (Promega, Madison, WI, USA) using standard molecular cloning techniques.

### Library screening and nucleotide sequencing

To obtain the cDNA in full length, the fragment of interest contained in the PCR clone (length in 582 base pairs) was used as a probe in the screening of human placental cDNA library (Clontech), using the standard filter hybridization techniques described (Wang et al, 1996, 1998). At the end of the third screening, we got 12 plaques containing the target nucleotide sequence. We sent the plaque which contained the longest DNA sequence for a test of its sequence on an ABI automated DNA

© 2001 Cancer Research Campaign sequencing system (Applied Biosystems, Foster City, CA), using the ABI Prism DNA sequencing kit with 2 different sets of primers. Multiple sequences were obtained in both orientations.

### Northern blot assay

40 μg of total RNA was used for Northern blot analysis (Wang et al, 1996, 1998). The probe of 2 μg HCCA2 full-length cDNA was labelled with 50 μCi α-$^{32}$P-dATP using Prime-a-Gene kit (Promega, Madison, WI, USA) according to the given protocol. The blot was exposed to X-ray film for 10 days at –80˚C. In order to calibrate relative quantities of loaded RNAs, the blot was rehybridized with a cDNA probe of the β-actin gene.

### Preparation of antisera

The C-terminal 160 amino acids (residues 1812 to 2292) were subcloned into the fusion expression vector pGEX-5X-1 (Pharmacia Biotech) and the sequence was verified by automated DNA sequencing. The fusion protein was purified as described (Wang et al, 1996, 1998) and used for immunizing rabbits at 2-week intervals. Antibody valence was determined by double immunodiffusion test and the specificity was confirmed by Western blotting.

### Expression of HCCA2 in transfected cells

The full-length HCCA2 cDNA was recloned into the pcDNA3 expression vector. When human embryonic kidney fibroblast 293 cells (ATCC CRL 1573) grew to nearly 70% confluency, the cells were transfected with 2 μg purified plasmid of HCCA2/pcDNA3 using Profection® Mammalian Transfection Systems (Promega, Madison, WI, USA), according to the manufacturer's instruction. Cells transfected with empty vector of pcDNA3 were served as control. 72 h after transfection, the cells were washed in phosphate-buffered saline and lysed in Triton-X 100 lysis buffer (Wang et al, 1996, 1998) at 4˚C. Cell lysates from HCCA2-transfected cells and control plasmid-transfected cells were separated on a 10% polyacrylamide gel, transferred to nitrocellulose membrane and probed with HCCA2 polyclonal antibodies (dilute in 1:2000) for 1 h.

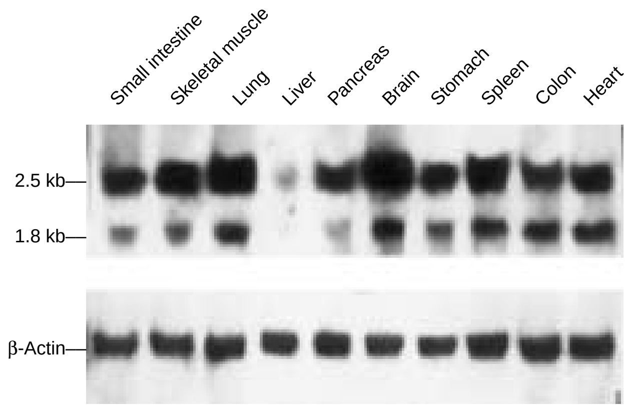

**Figure 1** Northern blot analysis of HCCA2 mRNA in human adult normal tissues. Upper panel showed HCCA2 mRNA was intensively expressed in human small intestine, muscle, lung, pancreas, brain, stomach, spleen, colon and heart tissue, but not expressed in liver tissues. The main transcript was 2.5 kb in length, whereas 1.8 kb transcript also could be observed. To show the relative quantities of loaded RNAs, the blot was rehybridized with β-actin cDNA probe (lower panel)

### Immunohistochemical staining for HCCA2

Surgical specimens including HCC tissues and surrounding liver tissues were fixed in 10% neutral formalin and embedded in paraffin. 5 μm paraffin sections were subjected to immunoperoxidase staining with HCCA2 polyclonal antibodies (dilutein 1:100) and Ki-67 antibody (Immunotech, Marseille, France) using Immunopure staining ABC kit (Dako, Glostrup, Denmark) according to the manufacturer's instruction. The sections were lightly counterstained with Harris's haematoxylin and examined under light microscope.

### Statistical analysis

We used the $\chi^2$ test or the Fisher's exact test for statistical analysis. A $P$ of less then 0.05 was considered significant.

### RESULTS

### Cloning and sequencing of HCCA2 cDNA

In DDPCR analysis, we identified a candidate band that was expressed preferentially in HCC. This fragment (length in 582 base pairs) was then subcloned into pGEM-T vector and served as specific probe to screen a human placental cDNA library. The full-length cDNA of 2520 base pairs (bp) was cloned from a human placental cDNA library. The cDNA sequence that was designated HCCA2 (hepatocellular carcinoma-associated gene 2) was submitted to EMBL/GenBank/DDBJ nucleotide sequence databases (Accession No. AF206328). HCCA2 contains a consensus initiation codon (Kozak, 1989) at position 891 followed by a single open reading frame of 1401 bp encoding 467 amino acids. The 3′ untranslated region of 202 bp had a consensus polyadenylation signal (AATAAA) beginning at the 19th base upstream of the poly (A) tail. Alignment at nucleotide and amino acid level showed no significant homologues with known genes. The deduced protein was estimated to be 50.65 kDa and had a pI of 8.14. Amino acid sequence analysis by GCG sequence analysis software package (version 9.1, Genetics Computer Group, Madison, Wisconsin) and PC/GENE (Version 5.03, Geneva University, Switzerland) showed that there were several putative modification sites. These include 2 N-glycosylation sites at amini acid of 71 and 441 and 6 N-myristoylation sites at amini acid of 70, 187, 285, 337, 408 and 435. Additionally, there were numerous phosphorylation sites for protein kinase: 2 cAMP- and cGMP-dependent protein kinase phosphorylation sites at amino acid of 127 and 248, 2 protein kinase C phosphorylation sites at 248 and 278 and 6 casein kinase II phosphorylation sites at 54, 154, 189, 346, 347 and 444. No transmembrane domain and signal peptide and nuclear targeting sequence were found. The HCCA2 protein was enriched in proline residues, and had 2 Src homology 3 (SH3)-binding domains (Cohen et al, 1995; Kang et al, 2000).

### Expression of HCCA2 mRNA in adult and fetal tissues

Northern blot analysis showed that HCCA2 mRNA appears to be widely expressed in adult tissues (Figure 1). The HCCA2 gene was showed to be intensively expressed in human small intestine,

© 2001 Cancer Research Campaign

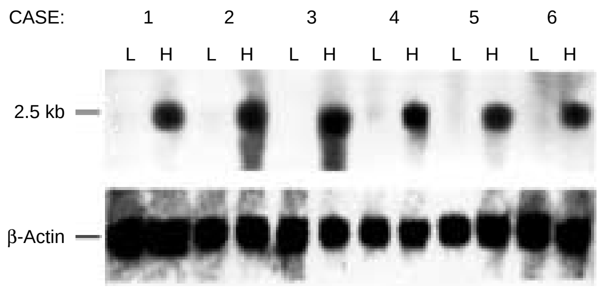

**Figure 2** Northern blot analysis of HCCA2 of paired HCC (H) and nontumour liver (L) tissues. A signal transcript of 2.5 kb was shown in 6 tumours (upper panel). Equal amount of total RNA loading as indicated by rehybridizing with β-actin cDNA probe (lower panel)

muscle, lung, pancreas, brain, stomach, spleen, colon and heart tissues, but not expressed in liver tissues. HCCA2 mRNA was detected in abundance in fetal liver (2 of 2 fetuses), lung (2 of 2 fetuses), brain (2 of 2 fetuses), and spleen (2 of 2 fetuses) (data not shown). Among all the adult and fetal tissues with positive signals, HCCA2 mRNA was observed in 2 transcripts: one was approximately 2.5 kb, which well corresponded to the size of the cloned cDNA, and the other about 1.8 kb.

### HCCA2 mRNA expression in HCC and its clinical significance

Among various adult tissues examined, HCCA2 mRNA was not detected in normal hepatic tissues. However, it intensively expressed in HCC tissues (Figure 2). HCCA2 mRNA expression was detected in 79% (34/43) of patients and was absent in 21% (9/43). All of them showed significantly high expression of HCCA2 mRNA in HCC tissues while no expression in surrounding liver tissues (except that HCCA2 mRNA was detected in low levels in only 2 cases of surrounding liver tissues). HCCA2 mRNA expression correlated closely with the invasion of liver cancer capsule ($P = 0.0007$) and the expression of Ki-67 protein ($P = 0.0022$), but that was not related to tumour size, tumour differentiation, serum AFP, HBsAg seropositivity, microscopic vascular cancer cell thrombi and adjacant small satellite nodules lesions ($P > 0.05$).

### Overexpression of HCCA2 in mammalian cells

After transfection, the full length of HCCA2 cDNA was transiently overexpressed in the human embryonic kidney fibroblast 293 cells. Using antiserum of HCCA2 for immunoblot analysis, a protein of approximately 50 kDa was recognized in transfected cells which neared the calculated size of 50.65 kDa. This band was not detected in cells transfected with an empty expression vector (Figure 3).

### Immunohistochemical assay

Immunostaining showed that HCCA2 protein was expressed in HCC tissues only, and it was localized in the cytoplasm (Figure 4). Among 43 patients examined, 11 (25.6%) revealed positive staining with HCCA2 antibody. The positive staining of Ki-67 protein was localized in the tumour cell nucleus, not expressed in surrounding liver. The positive expression rate of Ki-67 protein was 76.7% (33/43).

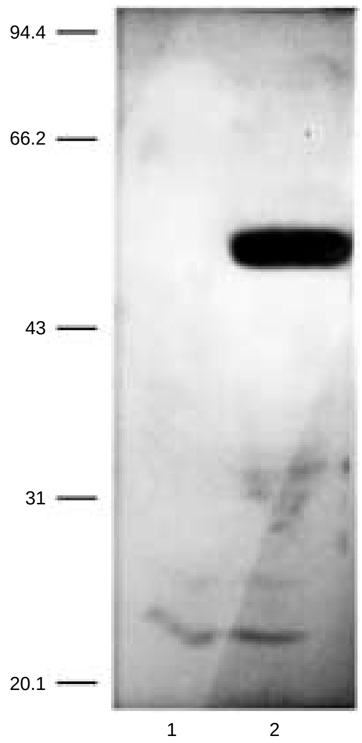

**Figure 3** Western blot analysis of HCCA2. Blot was probed with rabbit polyclonal antiserum against HCCA2. The antiserum of HCCA2 recognized a protein of approximately 50 kDa protein in human embryonic kidney 293 cells transfected with full-length HCCA2 expression construct (lane 2), but not in control plasmid-transfected cells (lane 1). Molecular size markers are indicated in kDa.

### DISCUSSION

Using a mRNA DD technique (Liang and Pardee, 1992; Liang et al, 1993), we have successfully isolated and characterized a full-length cDNA named HCCA2 with a transcript of 2520 bp (European Molecular Biology Laboratory/GenBank/DNA Data Bank of Japan accession no. AF206328). The HCCA2 mRNA appeared as a major transcript of 2.5 kb and encoded a putative

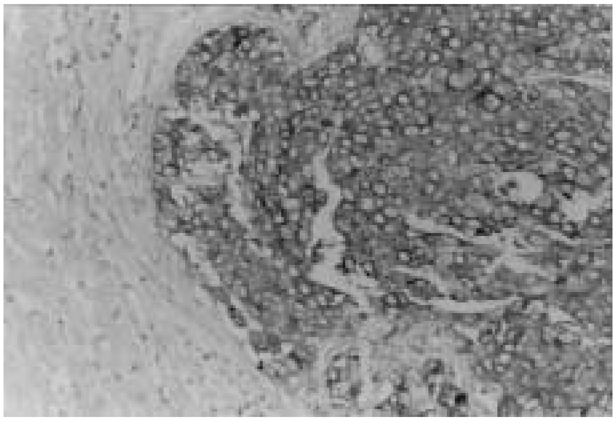

**Figure 4** Immunohistochemistry analysis showed that HCCA2 was expressed in hepatocellular carcinoma cells. The immunoreactivity oriented in liver cancerous cells and localized in cytoplasm. No signals were found in surrounding liver nontumour hepatocytes. Original magnification, ×300

protein of 467 amino acids containing no apparent signal peptide, transmembrane domain and nuclear targeting sequence. A smaller transcript of about 1.8 kb was identified in human adult and fetal tissues. The HCCA2 nucleotide and amino acid sequence showed no significant homologues with known genes. The HCCA2 protein is a proline-rich protein, because there were 62 prolines in 467 amino acids. The presence of 2 SH3 binding-domains indicates that the HCCA2 protein might have an important function in cell signal transduction (Cohen et al, 1995; Myllyharju and Kivirikko 1999; Nagasaki et al, 1999; Kang et al, 2000). The numerous phosphorylation sites may contribute to the transport of the HCCA2 protein (Graves and Krebs, 1999). The function of HCCA2 protein may also greatly depend on its phosphorylation status and SH3-binding domains. The immunohistochemical analysis showed that HCCA2 protein was located in the cytoplasm, indicating that HCCA2 is possibly a cytoplasmic protein. The predicted modification sites of the protein may be involved in diverse regulatory processes including intracellular signal transduction in the regulation of cellular proliferation. There is evidence that deregulation of protein phosphorylation is involved in several human cancers. HCCA2 as a possible oncoprotein may have functional abnormality and phosphorylative deregulation may be a mechanism.

Further studies on expression of HCCA2 may indicate its potential biological significance. We found that the HCCA2 mRNA was expressed widely in adult and fetal tissues, indicating that HCCA2 might be a normal developmentally regulated gene which was involved in the physical process of the distributed tissues. Other functions may also be involved. In normal adult tissues, HCCA2 was expressed in 2 transcripts of 2.5 kb and 1.8 kb, whereas in HCC, it was observed in a signal transcript 2.5 kb. We did not know the significance of these differences. Although HCCA2 mRNA was not detected in adult liver tissues, it was significantly expressed in HCC tissues, indicating that the HCCA2 may be stimulated to expression by some unknown factors in the process of liver oncogenesis. Also it implied that HCCA2 was regulated at transcript level in HCC. Because HCCA2 is a normal cellular gene, increased expression of HCCA2 in HCC may have genetic abnormality and act as an oncogene in the development of HCC. HCCA2 mRNA was expressed in abundance in fetal liver, but the levels became undetectable in adult liver, indicating that the expression of HCCA2 mRNA in adults was shut off and resurged in HCC. These findings suggest that HCCA2 is also an oncodevelopmental gene. In 43 patients with primary HCC, HCCA2 mRNA was detected in 34 (79%), who had significantly high expression in HCC tissues, but it was expressed in low levels in only 2 cases of cirrhotic surrounding liver tissues, suggesting that HCCA2 is a very common molecular event involved in the pathogenesis of HCC. These finding indicate that HCCA2 mRNA is overexpressed preferentially in HCC and can serve as a tumour biomarker for HCC. Of the 34 patients, 3 that had macroscopic portal vein tumour spread had significant expression of HCCA2 mRNA, implying that HCCA2 is a later incidence in HCC carcinogenesis.

To explore the potential biological role of HCCA2 mRNA expression, we selected 43 patients who had unicentric primary HCC. Of these, the expression of HCCA2 mRNA was found in 34 cases and correlated positively with the invasion of tumour capsule ($P$ = 0.0007) and the expression of ki-67 protein ($P$ = 0.0022) in HCC. It indicates that HCCA2 mRNA expression is a factor of HCC invasiveness and proliferation (Kaita et al, 1997; Tiniakos and Brunt, 1999). Ki-67 protein is a proliferating nuclear antigen present in replicating cells. The ability to document nuclear proliferation in the liver is essential to our understanding of hepatic regeneration and hepatocellular carcinoma (Kaita et al, 1997). Immunohistochemistry analysis further comfirmed in protein level that HCCA2 gene is intimately connected to HCC (Kaita et al, 1997). The positive rate of HCCA2 protein is significantly lower than the mRNA expression in tumour tissues, which may result from increased protease-mediated degradation rather than altered gene expression (Loda et al, 1997), or the expression level of HCCA2 protein is too low to be easily detected by immunohistochemistry method. Although the function of HCCA2 is still unknown, our results suggest that this novel gene codons for a cytoplasmic protein, and up-regulation of HCCA2 may play an important role in the development and/or progression of hepatocellular carcinoma. Hence, more studies are warranted to better understand the biological functions of the HCCA2 gene.

### ACKNOWLEDGEMENTS

We thank professor Gang Pei for kindly providing the anchored and arbitrary primers, Wenming Cong and Xiuzhong Zhang for pathological diagnosis and support with paraffin sections, Yi Hong, Jinzhang Zeng, Liang Tang, Xiuhua Qiu, Xiaobo Man for cooperation of some experiments and Zhengmin Miao for critical reading the manuscript. This work was supported by a grant from the Nation Natural Science Foundation (No 30000077 and 39825114), Chinese Human Genome Project and Scientific Finance of Chinese Postdoctor. The nucleotide sequence data reported in this paper will appear in the EMBL, Genbank and DDBJ nucleotide sequence databases under accession number AF206328.

### REFERENCES

Bain I and McMaster P (1997) Benign and malignant liver turnouts. *Surgery* **15**: 169–174

Chomczinski P and Sacchi N (1987) Single-step method of RNA isolation by acid guanidium thiocyanate-phenol-chloroform extraction. *Anal Bilchem* **162**: 156–161

Cohen GB, Ren R and Baltimore D (1995) Modular binding domains in signal transduction proteins. *Cell* **80**: 237–248

Duvoux C (1998) Epidemiology and diagnosis of HCC in cirrhosis. *Ann Chir* **52**: 511–517

Fujimoto Y, Hampton LL, Wirth PJ, Wang NJ, Xie JP and Thorgeirsson SS (1994) Alteration of tumor suppressor genes and allelic losses in human hepatocellular carcinoma in China. *Cancer Res* **54**: 281–285

Graves JD and Krebs EG (1999) Protein phosphorylation and signal transduction. *Pharmacol Ther* **82**: 111–121

Hui AM and Makuuchi M (1999) Molecular basis of multistep hepatocarcino-genesis: genetic and epigenetic events. *Scand J Gastroenterol* **34**: 737–742

Irene ON (1998) Molecular and cellular pathology of hepatocellular carcinoma. *J Gastronenterol Hepatol* **13**(suppl): S299–S303

Johnson RC (1997) Hepatocellular carcinoma. *Hepatogastroenterology* **44**: 307–312

Kaita KD, Pettigrew N and Minuk GY (1997) Hepatic regeneration in humans with various liver disease as assessed by Ki-67 staining of formalin-fixed paraffin-embedded liver tissue. *Liver* **17**: 13–16

Kang H, Freund C, Duke-Cohan JS, Musacchio A, Wagner G and Rudd CE (2000) SH3 domain recognition of a proline-independent tyrosine-based RKxxYxxY motif in immune cell adaptor SKAP55. *EMBO J* **19**: 2889–2899

Kondoh N, Wakatsuki T, Ryo A, Hada A, Aihara T, Horiuchi S, Goseki N, Matsubara O, Takenaka K, Shichita M, Tanaka K, Shuda M and Yamamoto M (1999) Identification and characterization of genes associated with human hepatocellular carcinogenesis. *Cancer Res* **59**: 4990–4996

Kozak M (1989) The scanning model for translation: an update. *J Cell Biol* **108**: 229–241

Liang P and Pardee AB (1992) Differential display of eukaryotic messenger RNA by means of polymerase chain reaction. *Science* **257**: 967–971

© 2001 Cancer Research Campaign

Liang P, Averboukh L and Pardee AB (1993) Distribution and cloning of eukaryotic mRNAs by means of differential display: refinements and optimization. *Nucleic Acids Res* **21**: 3269–3275

Loda M, Cukor B, Tam SW, Lavin P, Fiorentino M, Draetta GF, Jessup JM and Pagano M (1997) Increased proteasome-dependent degradation of the cyclin-dependent kinase inhibitor p27 in aggressive colorectal carcinomas. *Nat Med* **3**: 231–234

Myllyharju J and Kivirikko KI (1999) Identification of a novel proline-rich peptide-binding domain in prolyl 4-hydroxylase. *EMBO J* **18**: 306–312

Nagasaki K, Manabe T, Hanzawa H, Maass N, Tsukada T and Yamaguchi K (1999) Identification of a novel gene, LDOC1, down-regulated in cancer cell lines. *Cancer Lett* **140**: 227–234

Ozturk M (1999) Genetic aspects of hepatocellular carcinogenesis. *Semin Liver Dis* **19**: 235–242

Schlott T, Ahrens K, Ruschenburg I, Reimer S, Hartmann H and Droese M (1999) Different gene expression of MDM2, GAGE-1, -2 and FHIT in hepatocellular carcinoma and focal nodular hyperplasia. *Br J Cancer* **80**: 73–78

Sherman M (1995) Hepatocellular carcinoma. *Gastroenterology* **3**: 55–66

Tiniakos DG and Brunt EM (1999) Proliferating cell nuclear antigen and Ki-67 labeling in hepatocellular nodules: a comparative study. *Liver* **19**: 58–68

Wallner I, Hartmann H and Ramdori G (1994) Current therapeutic strategies in HCC, Part 1. *Leber Magen Darm* **24**: 150–154

Wang HY, Lian ZR, Lerch MM, Chen ZJ and Ullrich A (1996) Characterization of PCP-2, A Novel Receptor Protein Tyrosine Phosphatase of the MAM Domain Family. *Oncogene* **12**: 2555–2562

Wang HY, Fuchs M, Ciossek T, Chen ZJ and Ullrich A (1998) MAM-Subfamily Protein Tyrosine Phosphatases during Mouse Development. *Mechanisms of Development* **70**: 91–109

Xu GW, Sun ZT, Forrester K, Wang XW, Coursen J and Harris CC (1996) Tissue-specific growth suppression and chemosensitivity promotion in human hepatocellular carcinoma cells by retroviral-mediated transfer of the wild type p53 gene. *Hepatology* **24**: 1264–1268

Zhang H, Zhang R and Liang P (1996) Differential screening of gene expression difference enriched by differential display. *Nucleic Acids Res* **24**: 2454–2455

*© 2001 Cancer Research Campaign*
